# Data supporting the role of Fyn in embryonic sciatic nerve fasciculation

**DOI:** 10.1016/j.dib.2017.02.042

**Published:** 2017-02-22

**Authors:** Tomohiro Torii, Yuki Miyamoto, Kazuko Kawahara, Akito Tanoue, Yoichi Seki, Takako Morimoto, Masahiro Yamamoto, Junji Yamauchi

**Affiliations:** a Department of Neuroscience, Baylor College of Medicine, Houston, TX 77030, USA; b Laboratory of Molecular Neuroscience and Neurology, School of Life Sciences, Tokyo University of Pharmacy and Life Sciences, Hachioji, Tokyo 192-0355, Japan; c Laboratory of Molecular Pharmacology, Department of Pharmacology, National Research Institute for Child Health and Development, Setagaya, Tokyo 157-8535, Japan; d Tsumura Research Laboratories, Tsumura & Co., Inashiki, Ibaraki 200-1192, Japan

**Keywords:** Fyn, Knockout, Sciatic nerve, Fasciculation, Branching

## Abstract

Fyn is the cytoplasmic tyrosine kinase that has critical roles in many aspects of biological functions. In the central [1] and peripheral nervous systems [2], [3], Fyn plays the key role in initiating myelination by myelin-forming glial cells (Schwann cells and oligodendrocytes). Herein we provide the data regarding the role of Fyn in fasciculation and branching of embryonic peripheral nerves.

**Specifications Table**

**Table:** 

Subject area	Biology
More specific subject area	Neurobiology, molecular and cellular neuroscience
Type of data	Figure
How data was acquired	Immunohistochemistry, immunocytochemistry, immunoblotting,
Data format	Raw data, analyzed data
Experimental factors	Fyn knockout mice were used for experiments.
Experimental features	Histochemical analysis, immunoblotting analysis
Data source location	Laboratory of Molecular Neuroscience and Neurology, Department of Life Sciences, Tokyo University of Pharmacy and Life Sciences, Tokyo, Japan
Data accessibility	Data is available with this article

**Value of the data**

• This data set is of value to the scientific community to need the information for the biological effect of a cytoplasmic tyrosine kinase.
• The data provide the valuable information for the role of a cytoplasmic tyrosine kinase in developing nervous systems.
• The data allow us to promote our understanding of how a cytoplasmic tyrosine kinase plays the role in the peripheral nervous system.

## 1 Data

The data shared in this article provide immunohistochemical analyses of embryonic sciatic nerves (peripheral nerves) of Fyn knockout mice. The data also provide immunocytochemical analyses of Fyn knockout mouse peripheral neurons.

## 2 Experimental design, materials and methods

### 2.1 Data of Fyn knockout mouse

**Fig. 1:**
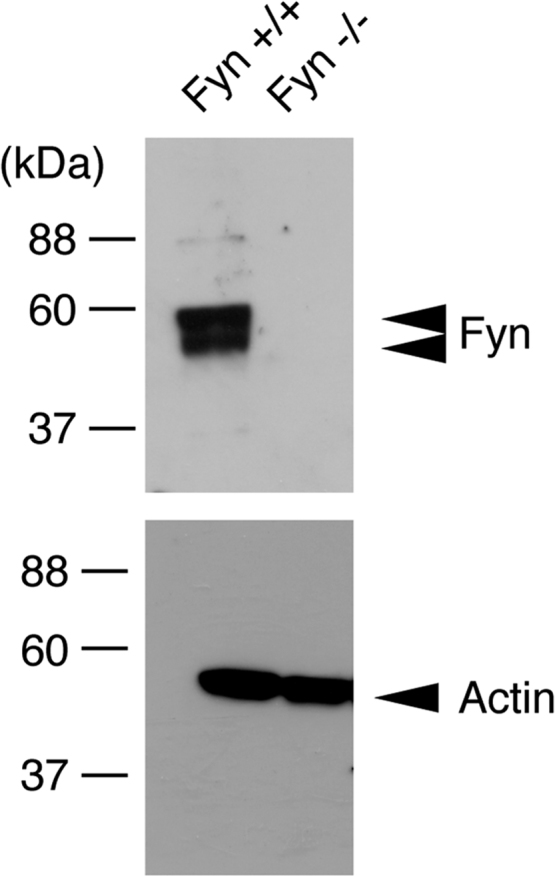
Immunoblotting of Fyn proteins using tissue lysates from Fyn knockout mice and the controls. The lysates from Fyn knockout mouse (Fyn−/−) and the control (Fyn+/+) sciatic nerves were used for immunoblotting with antibodies against Fyn and control actin. Fyn׳s double protein bands are predicted to be alternative splicing variants or degradation products.

**Fig. 2:**
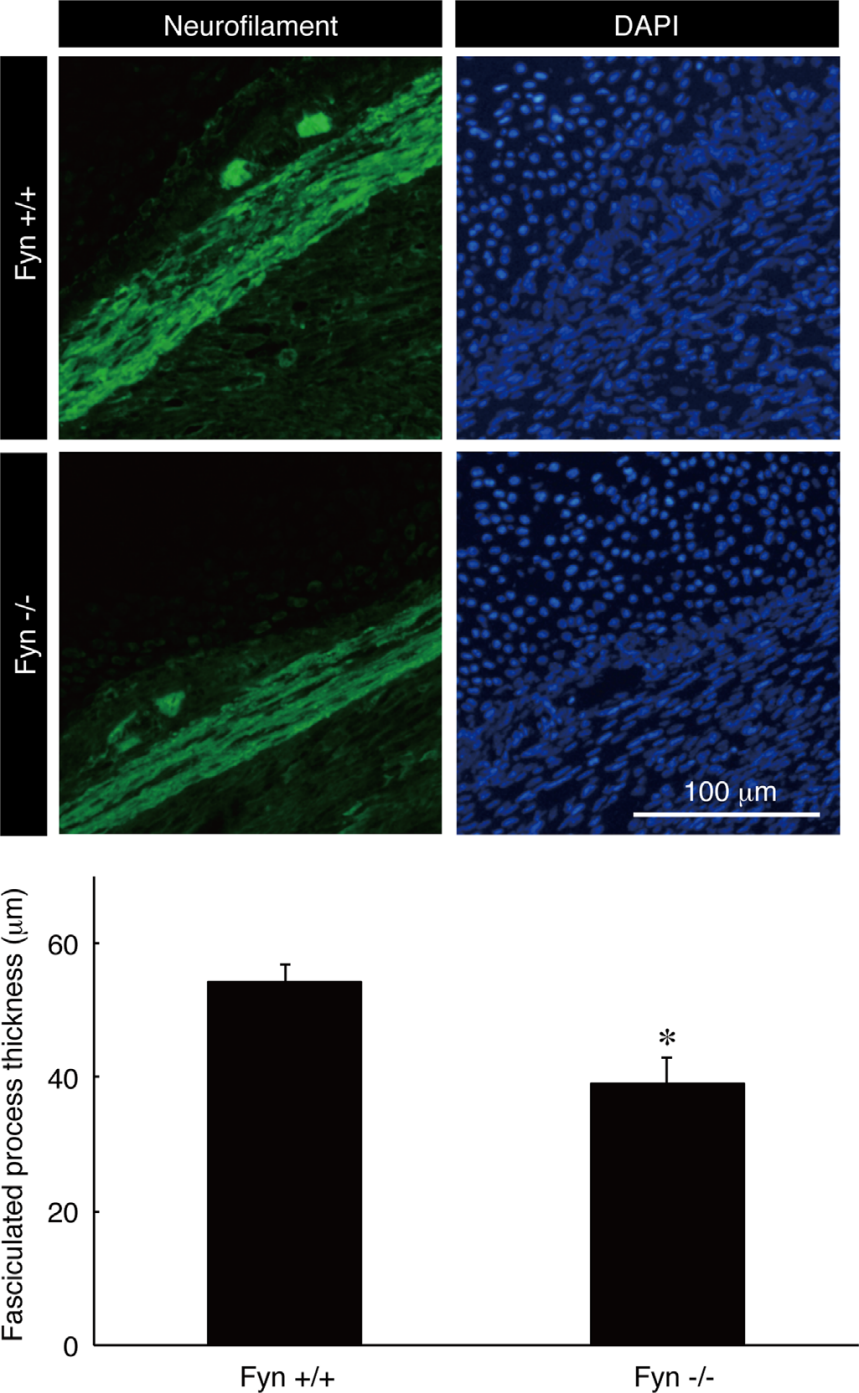
Staining of neurofilament proteins using longitudinal sections of Fyn knockout and the control sciatic nerves. Fyn knockout mouse (Fyn−/−) and the control (Fyn+/+) sciatic nerve longitudinal sections were used for staining with an anti-neurofilament antibody (green) and DAPI (blue). Fasciculated neuronal process thickness is also shown in the graph (**, p<0.01; n=6; Students’ *t*-test).

**Fig. 3:**
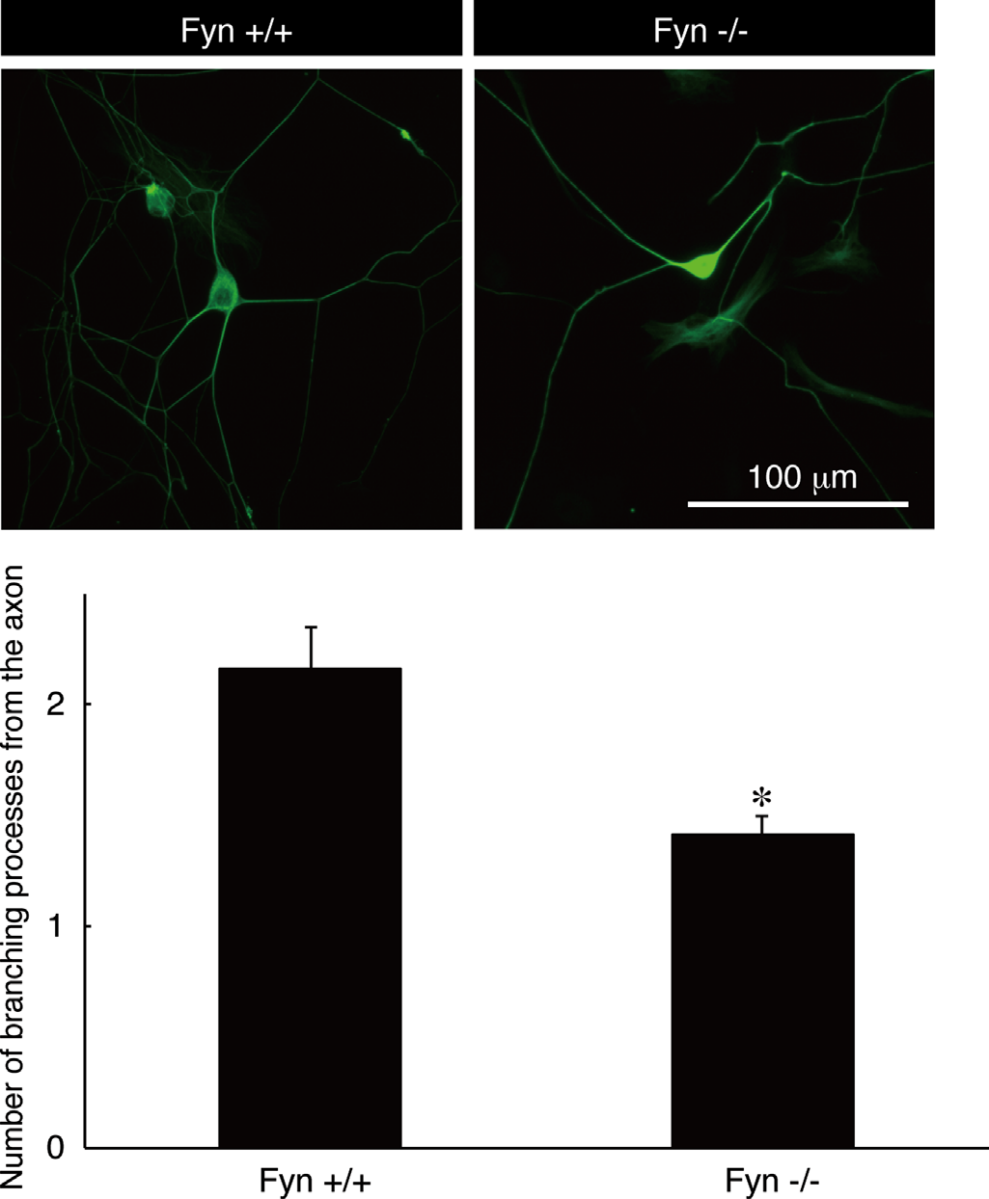
TUJ1 staining of primary DRG neurons from Fyn knockout and the control mice. Fyn knockout mouse (Fyn−/−) and the control (Fyn+/+) DRG neurons were used for staining with TUJ1 (green). The number of branching from the axon is also shown in the graph (**, p<0.01; n=6; Students’ *t*-test).

**Fig. 4:**
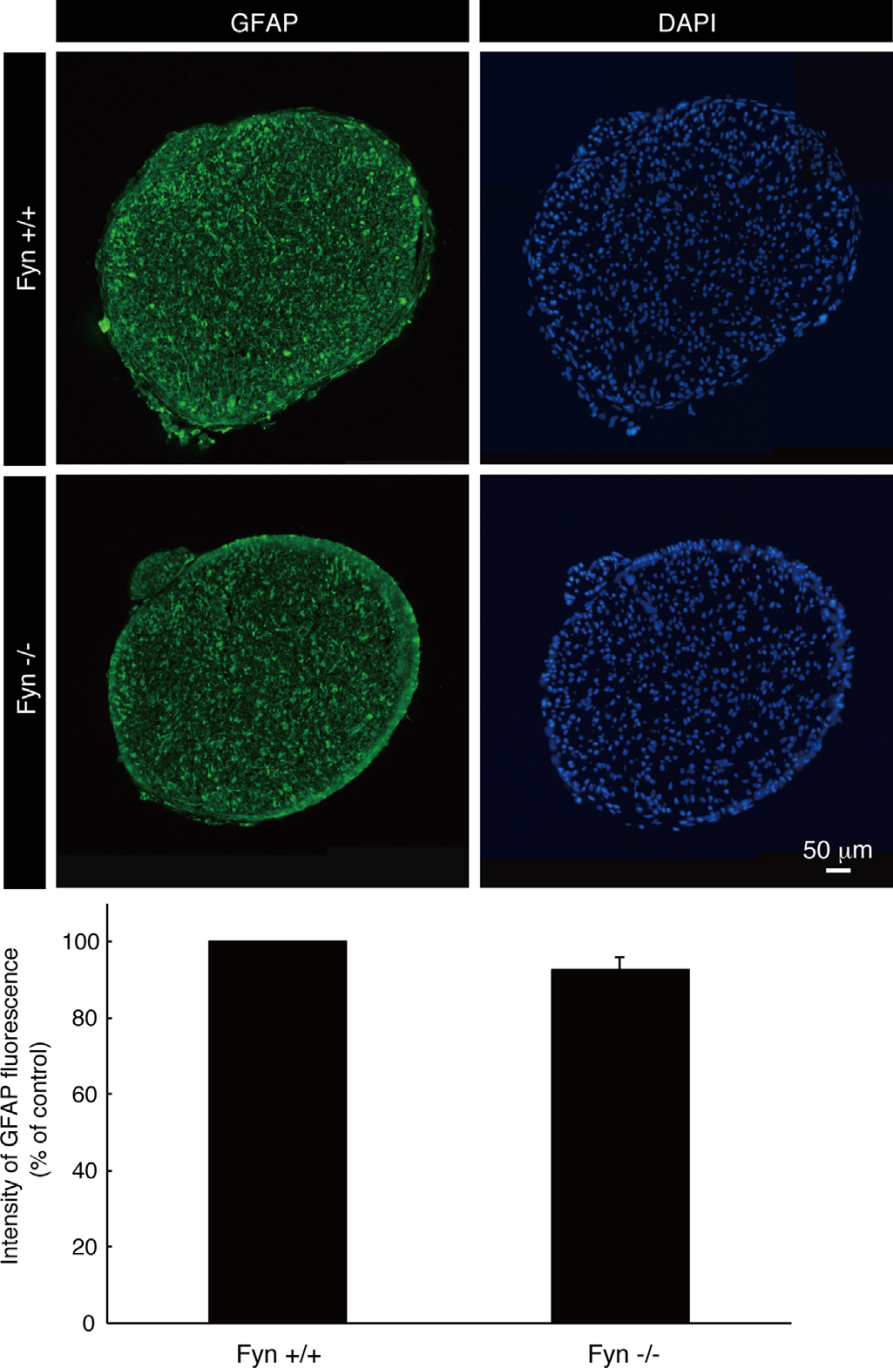
GFAP staining of cross sections of Fyn knockout and the control sciatic nerves. Fyn knockout mouse (Fyn−/−) and the control (Fyn+/+) sciatic nerve cross sections were used for staining with an anti-GFAP antibody (green) and DAPI (blue). Intensity of GFAP staining is also shown in the graph (n=3).

The tissue lysates from Fyn knockout mice [1], [2], [3] and the controls were immunoblotted with antibodies against Fyn and control actin (Fig. 1). Staining with an anti-neurofilament antibody and DAPI indicates fasciculation of embryonic sciatic nerves from Fyn knockout mice and the controls (Fig. 2). It is likely that the difference between Fyn knockout mice and the controls is more specific in the embryonic stage [4]. TUJ1 antibody staining indicates branching of primary peripheral dorsal root ganglion (DRG) neurons from Fyn knockout mice and the controls (Fig. 3). Staining with an anti-glial fibrillary acidic protein (GFAP) antibody and DAPI indicates the amounts of pro-myelinating Schwann cell cytoplasmic regions form Fyn knockout mice and the controls (Fig. 4).

### 2.2 Fyn knockout mouse

Cytoplasmic tyrosine kinase Fyn knockout mice (Stock Number: 002385) were obtained from the Jackson Laboratory (Hancock, ME, USA). Heterozygous offspring were mated with wild type C57BL/6JJms mice and the mutations were propagated in this strain for an additional 5 generations before it was crossed to produce experimental homozygotes. Genomic PCR was performed to identify respective knockout alleles according to the Jackson Laboratory׳s standard protocol. Male mice were used for experiments when gender was distinguishable. Knockout mice are fertile under experimental breeding conditions and apparently normal.

### 2.3 Immunoblotting

The lysates from mouse sciatic nerve tissues (embryonic day 18) were denatured and then separated on sodium dodecyl sulfate-polyacrylamide gels. The electrophoretically separated proteins were transferred to PVDF membranes, blocked with Blocking One reagent (Nacalai Tesque, Kyoto, Japan), and immunoblotted first with primary antibodies and then with peroxidase-conjugated secondary antibodies. The bound antibodies were detected using Nacalai Tesque׳s chemiluminescence reagent. Anti-Fyn and anti-actin (beta type) antibodies were from Atlas antibodies (Bromma, Sweden) and MBL (Aichi, Japan), respectively. At least three experiments were carried out under each condition, and a representative bot is shown in the figure.

### 2.4 Immunohistochemistry

Mouse sciatic nerve tissues (embryonic day 18) were perfused first with PBS and then with PBS containing 4% paraformaldehyde [5]. Subsequently, the tissues were postfixed with 4% paraformaldehyde, which was then replaced by 20% sucrose, and the tissues were embedded in Tissue-Tek reagent (Sakura Finetechnical, Tokyo, Japan). Microtome sections on glass slides were blocked using Blocking One reagent; subsequently, they were incubated with primary antibodies and then with fluorescence-labeled secondary antibodies. Glass slides were mounted using Vectashield reagent (Vector Laboratories, Burlingame, CA, USA). Fluorescent images were captured using a DM2500 microscope system (Leica) and were analyzed with LAS software (software attached to DMI2500, Leica) or captured with a BX51 microscope system (Olympus) and were analyzed with DP2-BSW software (software attached to BX51, Olympus). Anti-neurofilament and anti-GFAP antibodies were from Sigma-Aldrich (St. Louis, MO, USA) and BioLegend (San Diego, CA, USA), respectively. GFAP is the marker of pro-myelinating Schwann cells. At least three experiments were carried out under each condition, and a representative photograph is shown in each of the figures.

### 2.5 Immunocytochemistry

Primary mouse DRG neurons, which were isolated from DRGs (embryonic day 12.5) as described previously [5], were fixed with 4% paraformaldehyde. The fixed cultures were permeabilized with PBS containing 0.1% Tween-20 or 0.3% Triton X-100, blocked with Blocking One reagent, and then incubated first with primary antibodies and then with fluorescence-labeled secondary antibodies. The dishes were mounted with Vectashield reagent. The fluorescence images were captured with the fluorescence microscope system (DMI4000B; Leica, Wetzlar, Germany) and analyzed with AF6000 software (software attached to DMI4000B, Leica). TUJ1 was from BioLegend. TUJ1 is an antibody against neuronal fiber marker, tubulin beta3. At least three experiments were carried out under each condition, and a representative photograph is shown in the figure.
